# Parliament and the Professions

**Published:** 1920-01-10

**Authors:** 


### PARLIAMENT AND THE PROFESSIONS.

### Dispensers' Registration.

IN reply to a question put by Mr. Crooks, it was stated that the Pharmaceutical Society have passed a by-law, now awaiting the approval of the Privy Council, which provides for the registration as pharmaceutical chemists, or chemists and druggists, of assistants to apothecaries who obtained before December 21, 1908, a certificate of qualification under the Apothecaries' Act, 1815. It is understood that there is no possibility of a relaxation of the provisions of the by-laws in question in favour of such a case as that mentioned by Mr. Crooks, *i.e.*, a person who obtained the diploma of the Society of Apothecaries in 1910 and who since that date has had five years' Army service.

### Scarlet Fever.

DR. ADDISON stated that there were on December 18, 2,900 cases of scarlet fever in the institutions of the Metropolitan Asylums Board.

